# Supplementary figures and images for: Drinking and swimming around waterways: The role of alcohol, sensation-seeking, peer influence and risk in young people
Source: PLoS One. 2022 Nov 4;17(11):e0276558. doi: 10.1371/journal.pone.0276558 (PMC9635690; doi:10.1371/journal.pone.0276558)

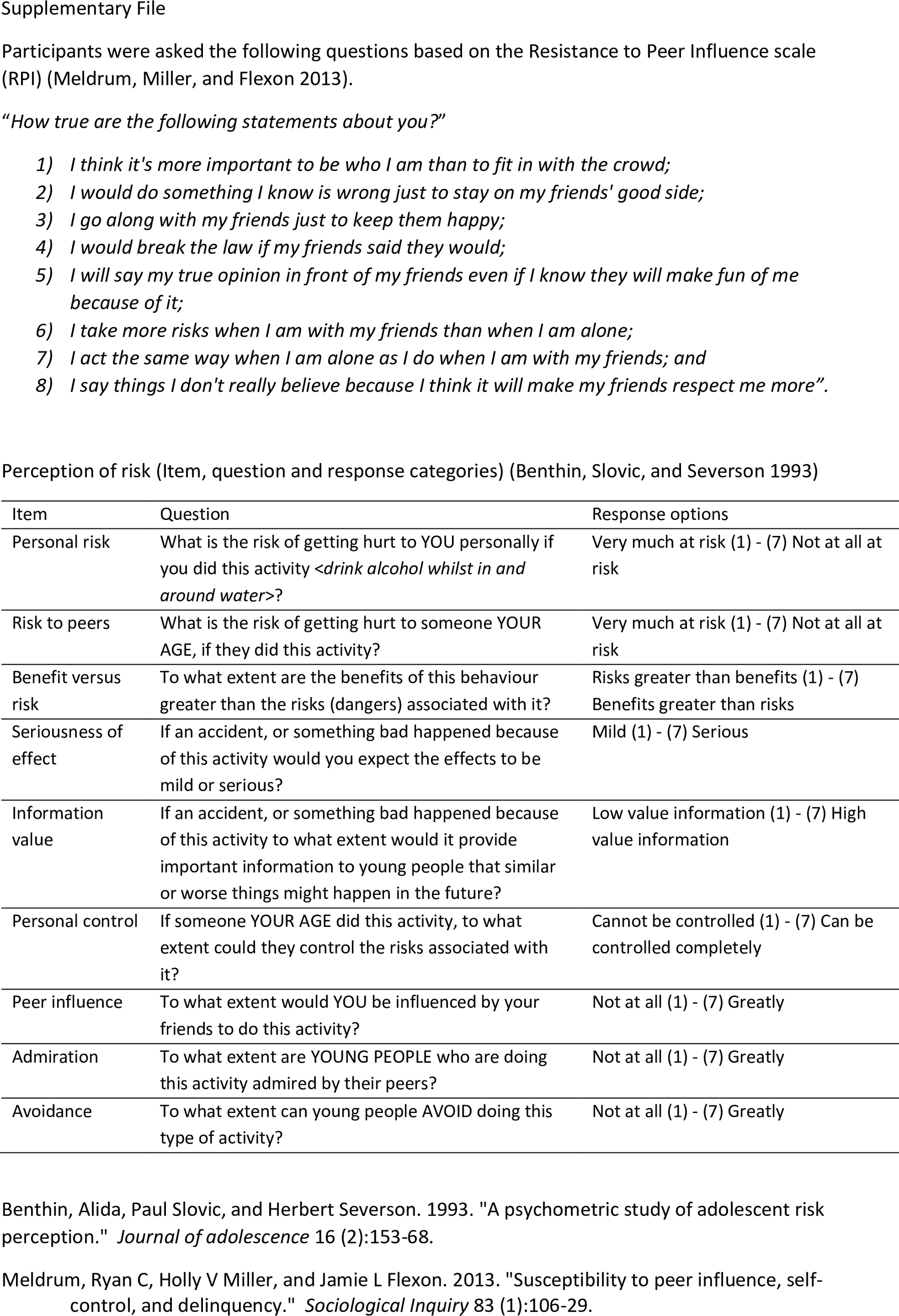

Supplement: S1 Table — (TIF) [file pone.0276558.s001.tif]

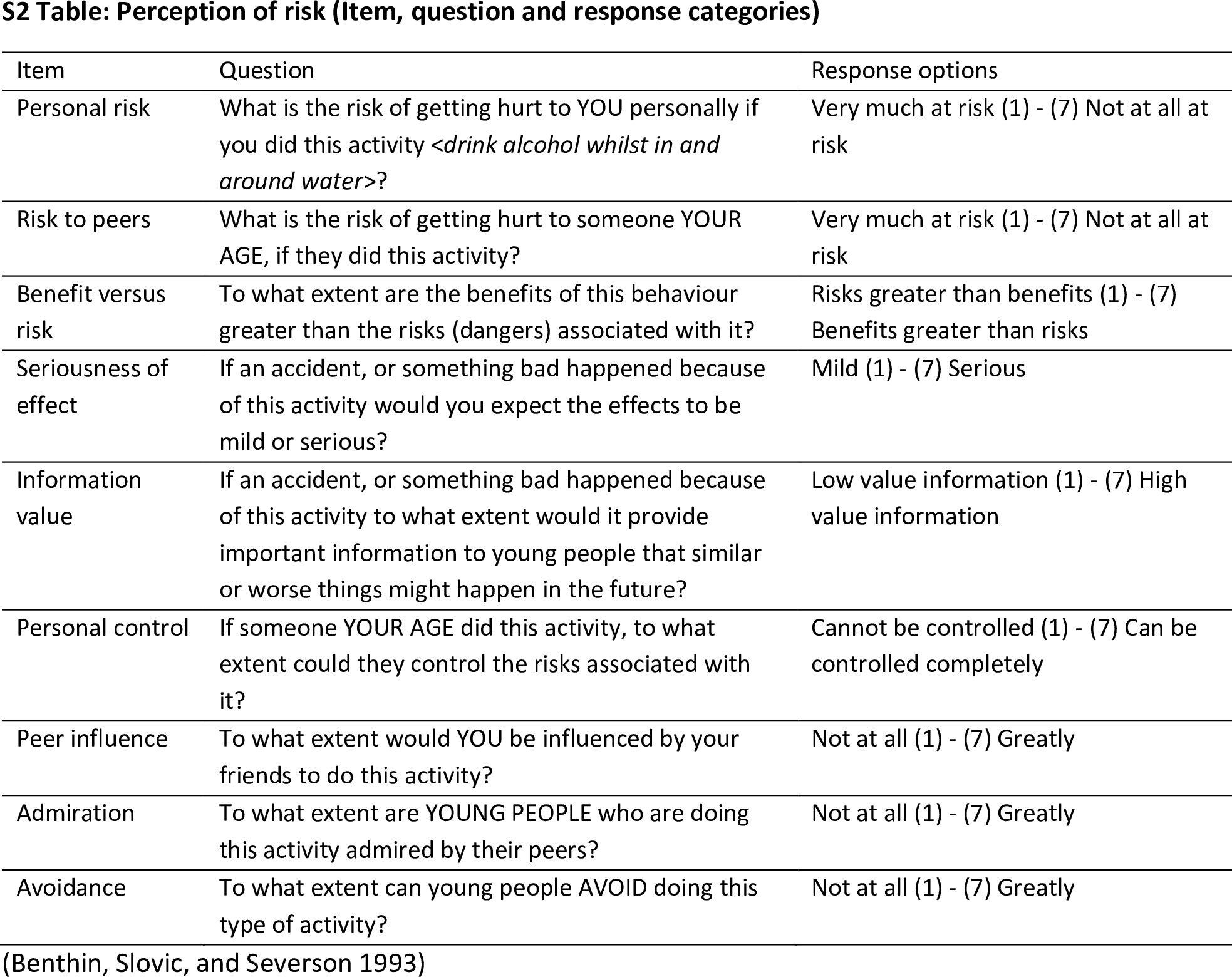

Supplement: S2 Table — (TIF) [file pone.0276558.s002.tif]
